# Expression of tissue factor and tissue factor pathway inhibitors during ovulation in rats: a relevance to the ovarian hyperstimulation syndrome

**DOI:** 10.1186/s12958-021-00708-1

**Published:** 2021-04-01

**Authors:** You Jee Jang, Hee Kyung Kim, Bum Chae Choi, Sang Jin Song, Jae Il Park, Sang Young Chun, Moon Kyoung Cho

**Affiliations:** 1 Animal Facility of Aging Science, Korea Basic Science Institute, Gwangju, 61186 Republic of Korea; 2 School of Biological Sciences and Biotechnology, Faculty of Life Science, Chonnam National University, Gwangju, 61186 Republic of Korea; 3 Center for Recurrent Miscarriage and Infertility, Creation and Love Women’s Hospital, Gwangju, 61917 Republic of Korea; 4 Department of Obstetrics and Gynecology, Chonnam National University Medical School, Gwangju, 61469 Republic of Korea

**Keywords:** Ovulation, Tissue factor, Tissue factor pathway inhibitor, Ovarian hyperstimulation syndrome

## Abstract

**Background:**

Blood coagulation has been associated with ovulation and female infertility. In this study, the expression of the tissue factor system was examined during ovulation in immature rats; the correlation between tissue factor and ovarian hyperstimulation syndrome (OHSS) was evaluated both in rats and human follicular fluids.

**Methods:**

Ovaries were obtained at various times after human chorionic gonadotropin (hCG) injection to investigate the expression of tissue factor system. Expression levels of ovarian tissue factor, tissue factor pathway inhibitor (Tfpi)-1 and Tfpi-2 genes and proteins were determined by real-time quantitative polymerase chain reaction (qPCR), and Western blot and immunofluorescence analyses, respectively. Expression levels of tissue factor system were also investigated in ovaries of OHSS-induced rats and in follicular fluid of infertile women.

**Results:**

The expression of tissue factor in the preovulatory follicles was stimulated by hCG, reaching a maximum at 6 h. Tissue factor was expressed in the oocytes and the preovulatory follicles. *Tfpi-2* mRNA levels were mainly increased by hCG in the granulosa cells whereas the mRNA levels of *Tfpi-1* were decreased by hCG. Human CG-stimulated tissue factor expression was inhibited by the progesterone receptor antagonist. The increase in *Tfpi-2* expression by hCG was decreased by the proliferator-activated receptor γ (PPARγ) antagonist. Decreased expression of the tissue factor was detected in OHSS-induced rats. Interestingly, the tissue factor concentrations in the follicular fluids of women undergoing in vitro fertilization were correlated with pregnancy but not with OHSS.

**Conclusions:**

Collectively, the results indicate that tissue factor and *Tfpi-2* expression is stimulated during the ovulatory process in rats; moreover, a correlation exists between the levels of tissue factor and OHSS in rats but not in humans.

**Supplementary Information:**

The online version contains supplementary material available at 10.1186/s12958-021-00708-1.

## Background

Ovulatory follicles undergo inflammation-like changes in response to the luteinizing hormone (LH) surge [1]. In rats, leukocyte infiltration in the periovulatory ovary [2] and extravasation of erythrocytes and fibrin clots in the follicular wall are observed during ovulation [3]. Fibrinogen secretion by bovine granulosa cells plays a role in ovulation by increasing the proteolytic activity [4]. Consistent with these observations, thrombin (a protease essential for fibrin formation) and its receptor are present in the periovulatory follicles in bovine [5, 6] and mouse [5] ovaries. In addition, the functional activity of thrombin and its receptor has been reported in human luteinized granulosa cells [7] and follicular fluid [8, 9]. These findings suggest the involvement of the blood coagulation system in the ovulatory process.

Tissue factor, a membrane-anchored glycoprotein, is the most important physiological regulator in thrombin generation and initiates the extrinsic pathway of coagulation via binding to factor VII [10]. The catalytic activity of the tissue factor-factor VIIa complex is inhibited by tissue factor pathway inhibitors (TFPIs), TFPI-1 and TFPI-2, belonging to the Kunitz family of serine protease inhibitors [11]. Tissue factor and TFPI-2 are detected in ovarian follicular fluid obtained from women undergoing in vitro fertilization [12]. Recently, it has been reported that TFPI-2 expression is stimulated by an ovulatory dose of gonadotropins in rat and human ovaries [13]. However, the detailed changes in the expression of the tissue factor system during the periovulatory period need to be assessed.

Factors regulating blood coagulation have been proven to be relevant to female infertility. Recurrent pregnancy loss is often related to increased levels of coagulant factors such as factor X and fibrinogen, and reduced levels of anticoagulant factors such as protein C [14]. The presence of blood clots within the cumulus matrix is associated with reduced blastocyte formation during in vitro fertilization in humans [15]. In addition, tissue factor acts as an important pro-inflammatory mediator in antiphospholipid antibody-induced pregnancy loss in mice [16]. Circulating tissue factor is elevated in women with polycystic ovary syndrome [17, 18]. Interestingly, TFPI-1 levels in blood, but not in follicular fluid, are significantly different between patients with ovarian hyperstimulation syndrome (OHSS) and non-OHSS patients [19].

OHSS is the most serious complication that, occurs during ovulation induction for the in vitro fertilization procedure [20]. The rat model of OHSS is established, demonstrating that vascular endothelial growth factor (VEGF) is a potential cause of the development of OHSS [21, 22]. Following treatment with human chorionic gonadotropins (hCG), an increase in VEGF concentration was observed in follicular fluid and serum in women undergoing in vitro fertilization [23]. Clinical manifestations of OHSS include massive extravascular fluid accumulation and hemoconcentration due to capillary leakage [20]. VEGF induces tissue factor expression in endothelial cells, increasing procoagulant properties of the vessel wall [24]. High tissue factor and low TFPI-1 levels in plasma were reported in patients with severe OHSS [25, 26]; however, no relationship was observed between follicular fluids of patients with and without OHSS [19]. Moreover, no report has yet elucidated the relationship between the tissue factor system and infertility factors, including OHSS in human follicular fluid.

Therefore, the present study was aimed to investigate the time- and cell-specific expression of tissue factor, TFPI-1 and TFPI-2 by gonadotropin treatment during the ovulatory process in rats. Moreover, as angiogenic factors play a role in the pathogenesis of OHSS [27], the relationship between the tissue factor system and OHSS was tested in the experimental model of OHSS in rats and in infertile patients undergoing in vitro fertilization.

## Materials and methods

### Hormones and reagents

Equine chorionic gonadotropin (eCG/PMSG), human chorionic gonadotropin (hCG), and chemical inhibitors including indomethacin, nordihydroguaiaretic acid, GW9662 were purchased from Sigma (St. Louis, MO, USA). RU486 was purchased from Enzo Life Sciences, Inc. (Farmingdale, NY, USA).

### Animals for superovulation induction and administration of ovulation-inhibiting agents

Immature female Sprague-Dawley rats were purchased from Korea Basic Science Institute (Gwangju, Korea) and Samtako BioKorea (Seoul, Korea). They were housed in groups in a room with controlled temperature and photoperiod (10-h dark/14-h light; lights on from 0600 to 2000 h). The animals had ad libitum access to food and water. Immature rats (26 days old; body weight, 60–65 g) were s.c. injected with 10 IU of eCG to induce multiple follicle growth. Two days later, some eCG-primed rats were i.p. injected with 10 IU hCG to induce superovulation. All animals were maintained and treated in accordance with the National Institutes of Health Guide for the Care and Use of Laboratory Animals, as approved by the Institutional Animal Care and Use Committee at Chonnam National University.

Five eCG-primed rats for each treatment group were i.p. injected 30 min before hCG administration with ovulation-inhibiting agents including progesterone receptor antagonist (RU486, 10 mg/kg body weight), cyclooxygenase inhibitor (indomethacin, 10 mg/kg body weight), lipoxygenase inhibitor (nordihydroguaiaretic acid, 3 mg/kg body weight), or proliferator-activated receptor γ (PPARγ) antagonist (GW9662, 2 mg/kg body weight) [28]. Six hours after hCG injection, the rats were euthanized using CO2 administration method and ovaries, upon removal of oviduct and fat pad, were collected for RNA isolation.

### Preparation of the rat model of ovarian hyperstimulation syndrome (OHSS)

**Fig. 1:**
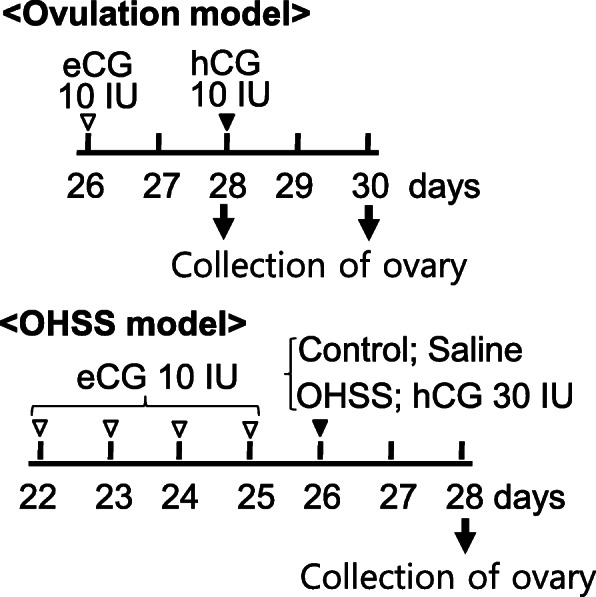
Experimental models showing conventional superovulation and OHSS induction procedures in rats

To prepare the OHSS rat model, immature rats (22 days old) were s.c. injected with 10 IU eCG at 0900 for four consecutive days to promote follicular development; this was followed by an i.p. injection of 30 IU hCG on the 5th day (on the 26th day of life) to induce OHSS (Fig. 1). As the control, rats were injected with 0.9% saline instead of hCG on the 5th day. Manifestation of OHSS includes the increased ovarian weight, VEGF expression and vascular permeability 48 h after hCG administration [22]. Subsequently, the rats were euthanized 48 h after hCG administration (on the 28th day of life); then, the ovaries were collected for RNA isolation. Ovaries were also collected from rats that were stimulated for superovulation in a routine manner 0 h and 48 h after hCG administration.

### Collection of ovaries and isolation of granulosa and theca cells of preovulatory follicles

Ovaries were collected from immature rats at different time points (0, 3, 6, 9 and 12 h) after eCG/hCG administration for RNA and protein detection of tissue factor, TFPI-1 and TFPI-2. For the isolation of the granulosa and theca cells of preovulatory follicles, the ovaries were incubated in DMEM/Ham’s F-12 medium (Gibco, Grand Island, NY, USA) containing 0.5 M sucrose and 10 mM EGTA at 37°С for 30 min. The ovaries were then washed thrice with phosphate buffered saline (PBS), and flattened to a single layer to easily identify the preovulatory follicles using fine forceps under a dissection microscope. The granulosa and theca cells were isolated from the preovulatory follicles using a 21-gauge needle for the measurement of mRNA levels.

### RNA isolation and real-time PCR analysis

To detect mRNA levels of tissue factor, TFPI-1 and TFPI-2 in ovaries and preovulatory follicles after hCG treatment (0, 3, 6, 9 and 12 h), total RNA was extracted using TRIzol reagent (Molecular Research Center, Inc., Cincinnati, OH, USA), according to the manufacturer’s instructions. Ten or twenty micrograms of total RNA was reverse-transcribed using the RevertAid M-MuLV reverse transcriptase kit (Fermentas, St. Leon-Rot, Germany) to evaluate gene expression. Real-time PCR was then performed on a Rotor-Gene Q 5plex (QIAGEN, Hilden, Germany), located at Korea Basic Science Institute (Gwangju, Korea), using the QuantiTect SYBR Green PCR Kit (QIAGEN) at 95°С for 20 s, 60°С for 20 s, and 72°С for 30 s. Specific primers were designed using the PRIMER3 software (Table 1). The average Ct value in triplicate for each gene was divided by the linear Ct value of *β-actin* to obtain relative abundance of the transcripts*. β-Actin* was used as an internal control for all measurements.

**Table 1:**
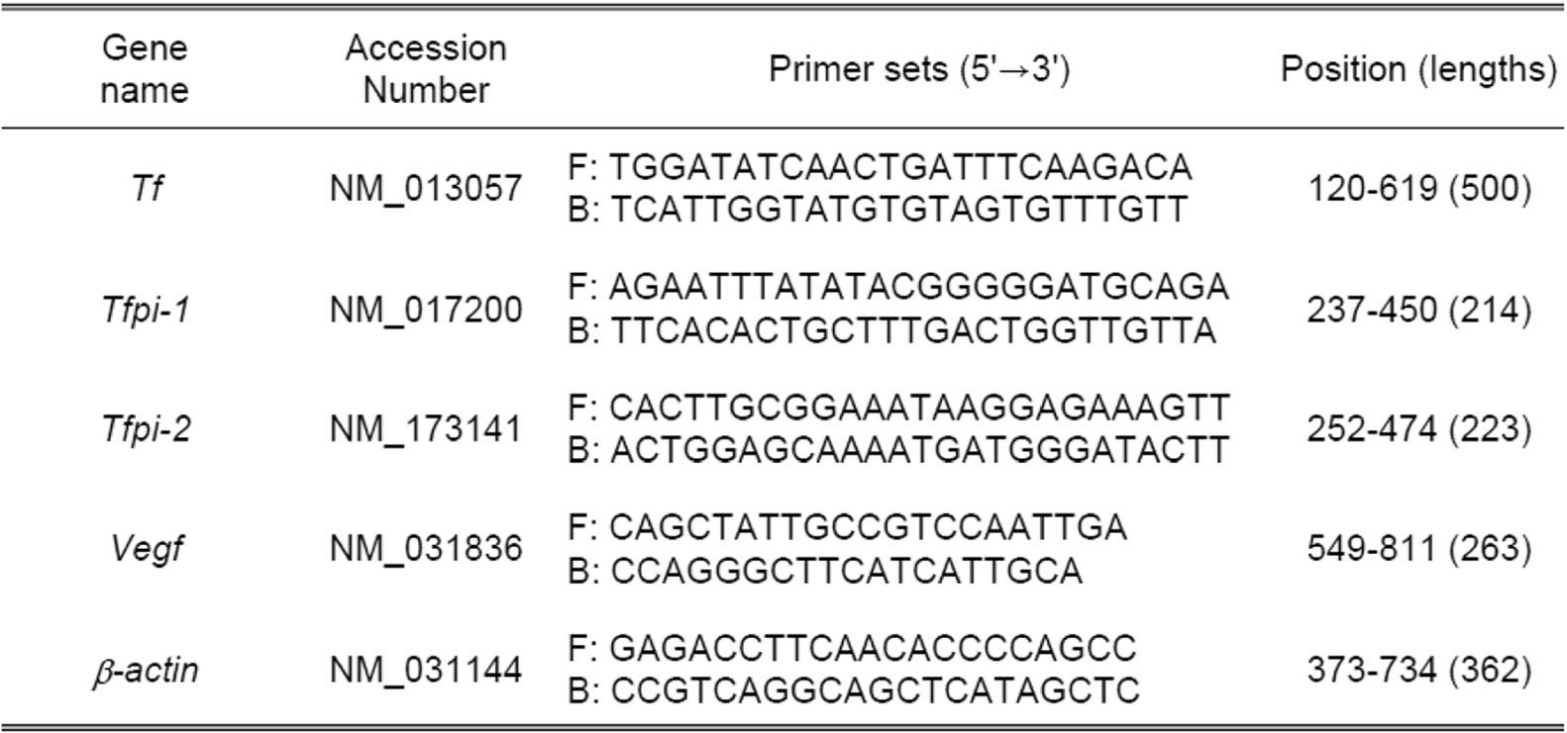
PCR primers used to obtain cDNAs for rat genes

*F* Forward, *B* Backward

### Western blot analyses

The ovarian lysates (30 μg) were resolved by 10% SDS-PAGE and transferred to nitrocellulose membranes (Amersham Bioscience, Arlington Heights, IL, USA), as previously described [3]. Briefly, the transferred membrane was blocked using 5% skim milk before immunoblotting using anti-tissue factor polyclonal antibodies (American Diagnostica, Inc., Stamford, CT, USA; 1:500 dilution) and horseradish peroxidase-conjugated secondary IgGs (1:1000 final dilution). Gapdh (Santa Cruz Biotechnology, Santa Cruz, CA, USA) was used as the loading control. Signals were visualized via enhanced chemiluminescence (Amersham Biosciences).

### Immunofluorescence

The localization of the tissue factor protein was determined by immunofluorescence as previously described [3]. Briefly, paraffin sections of ovary (5 μm thick) were incubated with 10% normal horse serum in PBS for 30 min to block non-specific binding of the antibody. The ovarian sections were probed with primary anti-tissue factor antibodies (American Diagnostica, Inc., 1:500 dilution) overnight and, then, washed thrice with PBS, followed by incubation with AlexaFluor 633 fluorescence antibodies (Invitrogen, Carlsbad, CA, USA; 1:500 dilution) for 1 h. After washing thrice with PBS, the sections were mounted on slides and the nuclei were stained with 4′, 6-diamidino-2-phenylindole (DAPI) in ProLong Gold Antifade reagent (Invitrogen). Digital images were captured using a TCS SP5 AOBS laser-scanning confocal microscope (Leica Microsystems, Heidelberg, Germany), located at the Korea Basic Science Institute Gwangju center.

### Collection of follicular fluid from women undergoing in vitro fertilization (IVF) and measurement of tissue factor concentrations via enzyme-linked immunosorbent assay (ELISA)

Follicular fluid was collected from 80 patients undergoing ovarian stimulation for IVF. Characteristics of patients based on the cause of infertility were presented in Supplemental Table 1. Forty-nine patients with infertility due to male (*n* = 22) or tubal factors (*n* = 27) served as controls. The male infertility patients were described as total motile count of < 10 million sperms/ml or normal morphology in < 4% of the sperm by strict criteria. Five women showed mild signs of OHSS after hCG administration during the IVF procedure. The causes of infertility among five OHSS patients include unknown factor (*n* = 3), oocyte donor and tubal factor. The inclusion criteria were age 21–42 years and normal uterine cavity on hysteroscopy. Patients who presented allergy to gonadotropins or other medications used in the treatment, or abusive use of any medications during the treatment were excluded. Our research was approved by the Institutional Review Board of Creation & Love Women’s Hospital (CLWH-IRB-2009-001).

Only clear follicular fluid, without blood or flushing medium contamination, was processed. After oocyte transfer, the follicular fluid (≈10 mL) aspirated from each patient was centrifuged for 10 min at 500×*g*. Supernatants of the follicular fluid samples were stored at − 80°С until the tissue factor concentrations were determined using an ELISA kit (EIAab Science Co., Wuhan, China). All the procedures were carried out according to the manufacturer’s instructions. Concentrations of tissue factor were detected in follicular fluids obtained from women with different infertility factors.

### Statistics

Statistical analyses were performed using the statistical software GraphPad Prism 5 (GraphPad Software, Inc. La Jolla, CA, USA). Data obtained from rat ovaries were presented as the means ± SEM. One way ANOVA, followed by Dunnett’s test, was used for comparisons among multiple groups. Comparisons between any two points were evaluated using Student’s two-tailed *t*-test. The levels of tissue factor in human follicular fluid were presented as the mean ± SD or median (range). Correlation analysis was performed using Spearman’s rho test. Pregnant and non-pregnant women were compared using the Kruskal-Wallis test or Mann–Whitney’s U-test. Fisher’s F-test was used to assess the relationship between two variables for parametric data. *P* < 0.05 was considered significant.

## Results

### Ovarian expression of tissue factor and Tfpi during ovulation in vivo

**Fig. 2:**
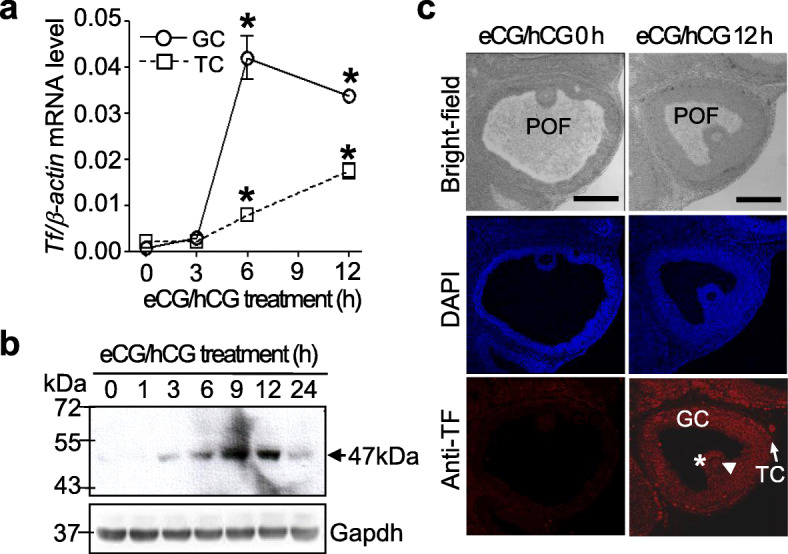
Stimulation of tissue factor (*Tf*) expression by eCG/hCG in ovarian preovulatory follicles. **a,** The level of tissue factor mRNA was detected in the isolated granulosa (GC) and theca (TC) cells of the preovulatory follicles using real-time PCR. Data are expressed as the mean ± SEM of three experiments. *, *P <* 0.05 vs. 0 h. **b**, Total lysates (30 μg protein/lane) extracted from the ovaries were analyzed by western blotting using anti-tissue factor polyclonal antibody (*n* = 4). Molecular weight is indicated to the *left* and the size of the tissue factor protein is indicated to the *right* using arrows. Protein loading was assessed using glyceraldehyde-3-phosphate dehydrogenase (Gapdh). **c**, Immunofluorescence analysis was performed to determine expression of the tissue factor protein in the preovulatory follicles. Fluorescence was analyzed by confocal microscopy after staining the samples with Alexa Flour 633 fluorescence antibodies (red color). Nuclei were stained with 4′, 6-diamidino-2-phenylindole (DAPI). Data are representative of four independently performed experiments. Arrowhead, Oocyte; asterisk, cumulus cells; arrow, theca cells; POF, preovulatory follicle; GC, granulosa cells; TC, theca cells. Scale bar, 200 μm

To examine gonadotropin regulation, the total RNA extracted from the preovulatory follicles of ovaries at different time points after hCG treatment was analyzed using real–time RT-PCR. As shown in Fig. 2a, the levels of tissue factor mRNA reached a maximum at 6 h (56.9-fold vs. that at 0 h; *P* < 0.05) and slightly decreased at 12 h in the granulosa cells. The expression of tissue factor in the theca cells increased gradually until 12 h (7.9-fold vs. that at 0 h). Western blot analysis revealed that the tissue factor protein had a molecular weight of 47 kDa, probably indicating that the tissue factor protein lacked the cytoplasmic domain, identical to the full-length protein at the initiation of thrombin generation (Fig. 2b). The levels of tissue factor protein increased transiently, reaching a maximum at 9 h after hCG treatment (5.3-fold vs. that at 0 h; *P* < 0.05). Immunofluorescence analysis demonstrated that the tissue factor protein was found in both the granulosa and theca cells at 12 h after hCG treatment (Fig. 2c). Interestingly, hCG treatment for 12 h increased tissue factor expression in the cumulus cells (Fig. 2c*, asterisk*) as well as in oocytes (Fig. 2c*, arrowhead*). No specific signal was detected in ovarian sections that were treated with goat control antibodies (anti-IgG; data not shown).

**Fig. 3:**
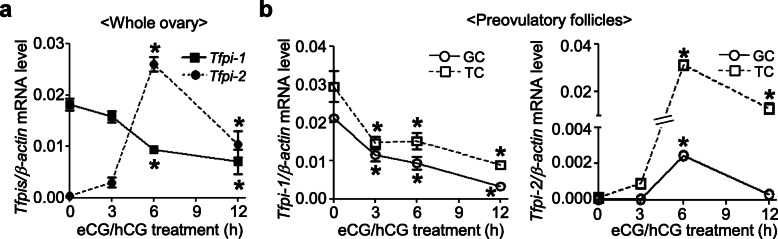
Changes in ovarian gene expression of *Tfpi-1* and *Tfpi-2* by eCG/hCG. Real-time PCR analysis was performed to determine the mRNA levels of *Tfpi-1* and *Tfpi-2* in the ovary **(a)** and in the granulosa (GC) and theca cells (TC) of the preovulatory follicles **(b)**. Data are presented as the mean ± SEM of three or four independently performed experiments. *, *P <* 0.05 vs. 0 h

Gonadotropin regulation of tissue factor pathway inhibitor (Tfpi) expression was also examined using real-time RT-PCR analysis. The levels of ovarian *Tfpi-2* mRNA were stimulated, reaching a maximum at 6 h after hCG treatment (68.7-fold increases vs. 0 h) whereas the levels of *Tfpi-1* mRNA gradually decreased until 12 h after hCG treatment (Fig. 3a). The *Tfpi-1* gene was expressed in both the granulosa and theca cells, with a gradual decrease in expression after hCG treatment (Fig. 3b, *left panel*). However, although the granulosa cell expression of *Tfpi-2* showed a transient stimulation at 6 h (18.8-fold vs. 0 h), the levels of *Tfpi-2* in the thecal cells were greatly increased, reaching a maximum at 6 h after hCG treatment (236.8-fold vs. 0 h; *P* < 0.05) (Fig. 3b, *right panel*).

### Regulation of tissue factor and Tfpi expression by ovulation-inhibiting agents in vivo

**Fig. 4:**
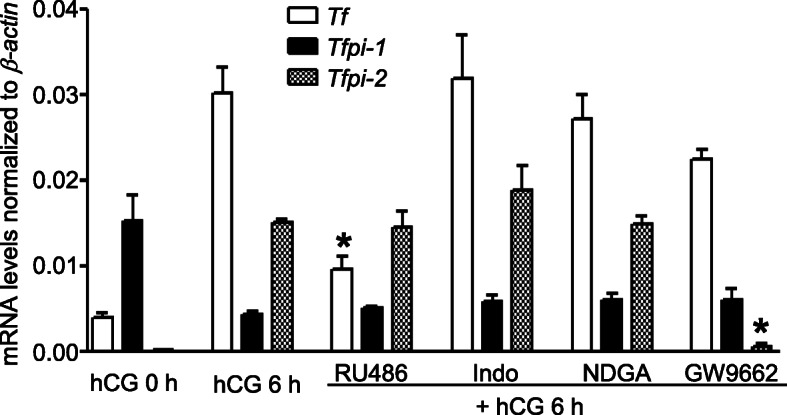
Changes in the ovarian gene expression of tissue factor, *Tfpi-1*, and *Tfpi-2*, by ovulation-inhibiting agents in vivo. Equine CG-primed immature rats were injected with vehicle (0.1% DMSO for control), progesterone receptor antagonist (RU486, 10 mg/kg), cyclooxygenase inhibitor (Indo; indomethacin, 10 mg/kg), lipoxygenase inhibitor (NDGA; nordihydroguaiaretic acid, 3 mg/kg), or PPARγ antagonist (GW9662, 2 mg/kg) 30 min before hCG administration. Ovaries were collected at 6 h for tissue factor (*Tf*) and ***Tfpi-2*** and, at 12 h, for ***Tfpi-1***, following hCG treatment for real-time PCR analysis. Data are presented as the mean ± SEM of five independently performed experiments. *, *P <* 0.05 vs. hCG 6 h or 12 h

To study the effect of ovulation-inhibiting agents on the expression of hCG-regulated tissue factor, *Tfpi-1*, and *Tfpi-2*, progesterone receptor antagonist (RU486), cyclooxygenase inhibitor (indomethacin), lipoxygenase inhibitor (nordihydroguaiaretic acid, NDGA), or PPARγ antagonist (GW9662) was administered 30 min before hCG stimulation in eCG-primed immature rats. Quantitative analysis using real-time PCR revealed that, at 6 h, the hCG-induced mRNA levels of tissue factor were inhibited by RU486 (68.1% inhibition; *P* < 0.05) but not the other agents (Fig. 4). The mRNA levels *Tfpi-1* were not affected by any inhibitor. Interestingly, injection with GW9662 significantly inhibited the hCG-induced *Tfpi-2* mRNA levels (96% inhibition vs. hCG at 6 h).

### Ovarian expression of tissue factor and Tfpi in the OHSS model in rats

**Fig. 5:**
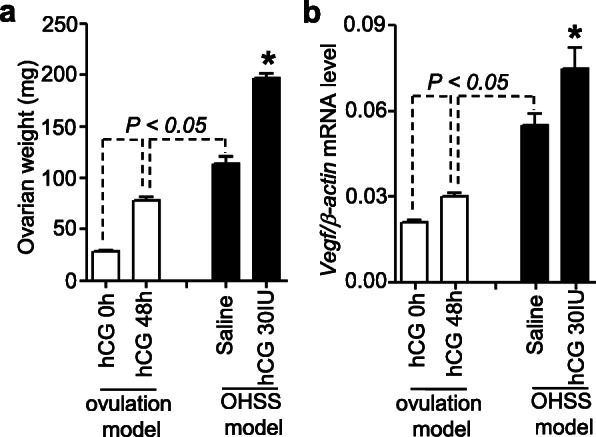
Increase in ovarian weight (**a**) and *Vegf* expression (**b**) in OHSS-induced rats. Ovaries were collected from OHSS-induced rats 48 h after saline or hCG administration to analyze *Vegf* expression using real-time PCR. Values are expressed as the mean ± SEM from six independently performed experiments. *, *P <* 0.05 vs. saline in the OHSS model

Blood clotting is related to OHSS [20]. Changes in the expression of tissue factor and TFPIs were therefore examined in a hormone-induced OHSS model in rats [22]. To validate the induction of OHSS in rats, the indexes for the occurrence of OHSS were examined. Ovarian weight was increased after hCG administration for 48 h in ovulation-induced rats (Fig. 5a). Ovarian weight was markedly increased in OHSS-induced rats upon administration of 30 IU of hCG for 48 h compared with that in rats treated with saline for 48 h. The ovarian levels of vascular endothelial growth factor (*Vegf*) were increased in ovulation- and OHSS-induced rats treated with hCG and saline, respectively, for 48 h (Fig. 5b). The levels of *Vegf* mRNA were higher (*P* < 0.05) in OHSS-induced rats administered with hCG than in those administered with saline. The vascular permeability was also higher in OHSS-induced rats administered with hCG than in those administered with saline indicating the elevation of capillary permeability (Supplemental Fig. S1). These results indicated the successful induction of OHSS in rats.

**Fig. 6:**
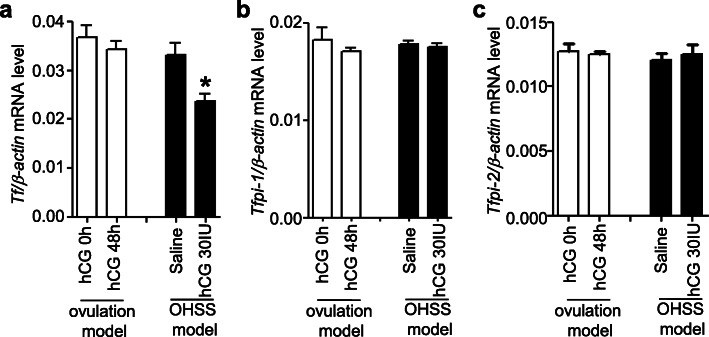
Expression of tissue factor (*Tf*; **a**), *Tfpi-1* (**b**), and *Tfpi-2* (**c**) in the OHSS-induced rats. Ovaries were collected from the ovulation- and OHSS-induced rats 48 h after saline or hCG administration to analyze the mRNA levels using real-time PCR. Data are presented as the mean ± SEM from four independently performed experiments. *, *P <* 0.05 vs. saline in the OHSS model

Although the ovarian expression of tissue factor was not changed by hCG in the ovulation model, the mRNA levels of ovarian tissue factor were significantly lower in OHSS-induced rats injected with hCG than in those injected with saline (Fig. 6a), suggesting that tissue factor can be a potential biomarker of OHSS in humans. The levels of ovarian *Tfpi-1* and *Tfpi-2* remained unaltered upon hCG administration in ovulation- or OHSS-induced rats (Fig. 6b and c).

### Detection of tissue factor in follicular fluid samples obtained from women undergoing in vitro fertilization (IVF)

**Fig. 7:**
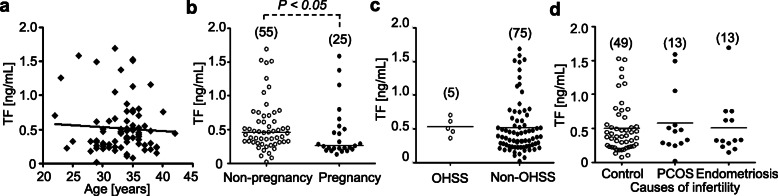
Tissue factor (TF) levels in the human ovarian follicular fluid of women undergoing the IVF procedure. Follicular fluids were collected from 80 women undergoing IVF. Levels of tissue factor were determined using ELISA. **a,** Correlation with age. Pearson correlation analysis was performed to evaluate the association between tissue factor levels and the patient’s age. **b**, Correlation with pregnancy. The data were analyzed by Mann Whitney *U* test. **c**, Correlation with OHSS. **d**, Correlation with infertile patients. Control group included patients with infertility due to male (*n* = 22) or tubal factors (*n* = 27). The data were analyzed by *F-* test. The scatter plot with bars represents the mean values of tissue factor levels. Numbers in parenthesis indicate the number of samples used. OHSS, Ovarian hyperstimulation syndrome; PCOS, polycystic ovary syndrome

As tissue factor expression was stimulated during ovulation and decreased in the OHSS rat model, the possibility of using tissue factor as a biomarker of female infertility was investigated by determining the amount of tissue factor in the follicular fluids of women undergoing IVF. No correlation was found between the tissue factor level and age of the women (Fig. 7a). Interestingly, the tissue factor levels in follicular fluids collected at oocyte retrieval were correlated with pregnant outcome. Infertile patients who became pregnant had a significant lower levels of tissue factor in follicular fluids at oocyte retrieval (447.6 ± 78.25 pg/mL) than those who did not become pregnant (547.2 ± 50.95 pg/mL) (Fig. 7b, *P* = 0.0301). Tissue factor levels were not different between OHSS (531.4 ± 59.38 pg/mL) and non-OHSS group (515.0 ± 45.49 pg/mL) (Fig. 7c). The correlation between tissue factor levels and the causes of infertility was also examined. Tissue factor levels were not different between control group (500.1 ± 52.59 pg/mL) and PCOS (580.0 ± 135.60 pg/mL) or endometriosis group (506.6 ± 114.40 pg/mL) (Fig. 7d).

## Discussion

Ovulation resembles the tissue remodeling process of blood coagulation. In this study, we report that tissue factor, an initiator of the extrinsic coagulation pathway, is induced during ovulation in rats. We also report that tissue factor expression is correlated with OHSS in rats and humans, which is characterized by an excessive response to ovulation-inducing hormones as well as massive hemoconcentration [20]. The expression of TFPI-1 was decreased by hCG, suggesting the potentiation of the tissue factor activity. Furthermore, the hCG-mediated stimulation of tissue factor expression in the granulosa cells of the preovulatory follicles required progesterone receptor activation. As the progesterone receptor is the key transcription factor inducing follicular rupture [29], the tissue factor gene, as a downstream gene for the progesterone receptor, may be involved in follicular rupture via formation of a fibrin clot after the release of fertilizable oocyte [30]. In contrast to TFPI-1, TFPI-2 expression was stimulated during ovulation. The increased expression of TFPI-2 mediated by hCG, observed in human and rat preovulatory follicles, may play a role in the tissue remodeling process that occurs during follicular rupture [13].

It is likely that tissue factor produced by the granulosa cells is the major coagulation factor during follicular rupture. The ovulatory surge of LH progressively triggers an elevation in ovarian blood flow and vascular permeability followed by ovarian hyperemia, edema, and extravasation of blood in preovulatory follicles, ultimately resulting in the rupture of the follicular wall [1]. Tissue factor was produced 9–12 h after LH/hCG administration, indicating that tissue damage during follicular rupture may trigger the expression of tissue factor. Follicular rupture occurs about 12 h after the LH surge in rodents. Tissue factor may play a role in repairing the damaged follicular wall via formation of a fibrin clot after the release of the oocyte into the oviduct. The present observation, in which the tissue factor gene is a downstream gene for the progesterone receptor, supports the hypothesis that tissue factor may be the major ovarian coagulation factor during periovulatory tissue remodeling. Studies on the targeted deletion of the progesterone receptor gene in mice indicate that the progesterone receptor is specifically and absolutely required for the rupture of the preovulatory follicle and oocyte release [31]. Tissue factor was also expressed in the cumulus cells and oocytes. The fact that the presence of blood clots in the human cumulus-oocyte complex was associated with reduced oocyte quality and blastocyst formation [15] indicates that tissue factor expressed in the cumulus cells and oocytes may be required for post-fertilization development.

Tissue factor may stimulate angiogenesis in the corpus luteum by inducing VEGF expression. The development of the corpus luteum is accompanied by rapid angiogenesis with the comparable rates of vascular formation in the growing tumors [32]. VEGF is the most remarkable regulator of angiogenesis in the corpus luteum [32, 33]. Of note, tissue factor, apart from its essential role in the coagulation process, exerts a role in angiogenesis in the tumor [34], possibly via release of VEGF [35]. Because the corpus luteum secrets progesterone to maintain intrauterine pregnancy [36], the present observation of correlation between levels of tissue factor and pregnancy may reflect a role of tissue factor in the function of corpus luteum by stimulating angiogenesis via VEGF.

Tissue factor could be used as a marker for OHSS. Several mediators involved in ovulation have been proposed as factors leading to OHSS such as estrogens, histamine, prostaglandins, cytokines [27] and the renin-angiotensin [37]. Vascular endothelial growth factor (VEGF) has also been implicated as a prime causative factor of OHSS progression. Levels of VEGF in serum and follicular fluid may predict the occurrence, severity, and progression of OHSS [23, 38]. In our study, an increase in the ovarian expression of *Vegf* was observed in the OHSS-induced rats. Using this OHSS model, a decrease in the ovarian expression of tissue factor was observed in OHSS-induced rats, suggesting that tissue factor may be one of the indicators for the occurrence of OHSS. Changes in the hemostatic system have been reported to be responsible for an increased thrombotic risk in patients with OHSS [20].

Although the tissue factor levels were correlated with OHSS in rats, we could not observe the correlation between tissue factor levels in follicular fluid and OHSS patients undergoing in vitro fertilization (IVF). However, an increase in tissue factor levels in the plasma has been reported in patients with severe OHSS [26]. These different outcomes may be attributed to the difference in samples, follicular fluid vs. plasma. The concentration of the tissue factor protein in human follicular fluid has been estimated to be 3.7-fold higher than that in the plasma [39]. In mammalian ovarian follicular fluid, only the tissue factor-dependent extrinsic pathway is present [8]; most tissue factors in follicular fluid must be generated locally by the granulosa cells of preovulatory follicles [39]. Additionally, it must be noted that the samples of human follicular fluid were obtained from women undergoing massive hCG stimulation during IVF. Therefore, depending on the measurement of tissue factor levels in plasma or follicular fluid, different outcomes between OHSS and non-OHSS patients might be produced. Decreased TFPI-1 levels have been reported in the plasma, but not the follicular fluid, of patients with OHSS [19]. Further studies are needed to confirm the possible use of tissue factor as a biomarker for OHSS using a large number of samples.

The lower levels of tissue factor in the follicular fluid collected at oocyte retrieval was observed in infertile women who became pregnant compared with those who did not become pregnant, suggesting the possible use of tissue factor as a pregnancy index. Pregnancy itself leads to a hypercoagulable state secondary to increased concentrations of coagulant factors [14]. Indeed, the expression of coagulation factors, including antithrombin and fibrinogen, is significantly decreased in the chorionic villi of patients with recurrent spontaneous abortion [9]. It is thus likely that coagulation factors play a role in maintaining a normal pregnancy. Tissue factor expression in neutrophils contributes to pregnancy loss induced by antiphospholipid antibodies in mice [16]. However, concentrations of tissue factor or TFPI-1 in the plasma of patients with OHSS are not correlated with the outcomes of pregnancy [26]. The present hypothesis of the predictive role of tissue factor as a pregnancy index should be assessed by ad hoc studies.

In contrast to the expression of tissue factor, TFPI-1 expression decreased continuously after LH/hCG administration, providing an environment for higher activity of tissue factor. The presence of TFPI-1 has been reported in human granulosa cells and preovulatory follicular fluid [39]. In contrast, TFPI-2 expression was markedly increased upon LH/hCG administration. Unlike TFPI-1, which inhibits the activity of tissue factor, the true function of TFPI-2 has not yet been clearly elucidated. TFPI-2 is involved in blood coagulation due to its ability to inhibit the formation of the tissue factor-factor VIIa complex [40, 41]. TFPI-2 also plays a role in remodeling the extracellular matrix by virtue of being a serine protease inhibitor [42]. TFPI-2 inhibits the protease activity of plasmin [43] and metalloproteinases [44]. Indeed, it has been demonstrated that TFPI-2 regulates ovulatory proteolysis by manipulating the activity of plasmin during the periovulatory period [13]. Therefore, TFPI-2 could have a role in modulating the remodeling of the extracellular matrix rather than modulating blood coagulation during the periovulatory period. As the PPARγ plays a role in tissue remodeling during ovulation [45], our finding that TFPI-2 expression was suppressed by a PPARγ antagonist supports this hypothesis.

## Conclusions

In summary, we have shown that tissue factor and TFPI-2 are induced in preovulatory follicles during the ovulatory process in rat ovaries and provide compelling evidence that tissue factor system can regulate the ovulatory process via progesterone receptor and PPARγ pathways. In addition, the levels of tissue factor are higher in ovaries of OHSS-induced rats supporting the hypothesis that tissue factor can be used as a biomarker for OHSS. The concentration of tissue factor in the follicular fluids was correlated with pregnancy of patients, but not with OHSS, undergoing IVF. Further investigation is needed on a large number of patients with infertility to determine the possible role of tissue factor as a marker for OHSS and pregnancy.

## Supplementary Information

**Additional file 1.**

## Data Availability

Not applicable.

## Abbreviations

eCG: Equine chorionic gonadotropin
hCG: ELISA, enzyme-linked immunosorbent assay; human chorionic gonadotropin
IVF: in vitro fertilization
LH: luteinizing hormone
OHSS: ovarian hyperstimulation syndrome
qPCR: real-time quantitative polymerase chain reaction
TFPI: tissue factor pathway inhibitor
VEGF: vascular endothelial growth factor
