# Supplementary material for: Expression of tissue factor and tissue factor pathway inhibitors during ovulation in rats: a relevance to the ovarian hyperstimulation syndrome
Source: Reprod Biol Endocrinol. 2021 Apr 1;19:52. doi: 10.1186/s12958-021-00708-1 (PMC8017805; doi:10.1186/s12958-021-00708-1)
Supplement: Supplementary file 1 — Additional file 1. [file 12958_2021_708_MOESM1_ESM.pptx]

## Slide 1
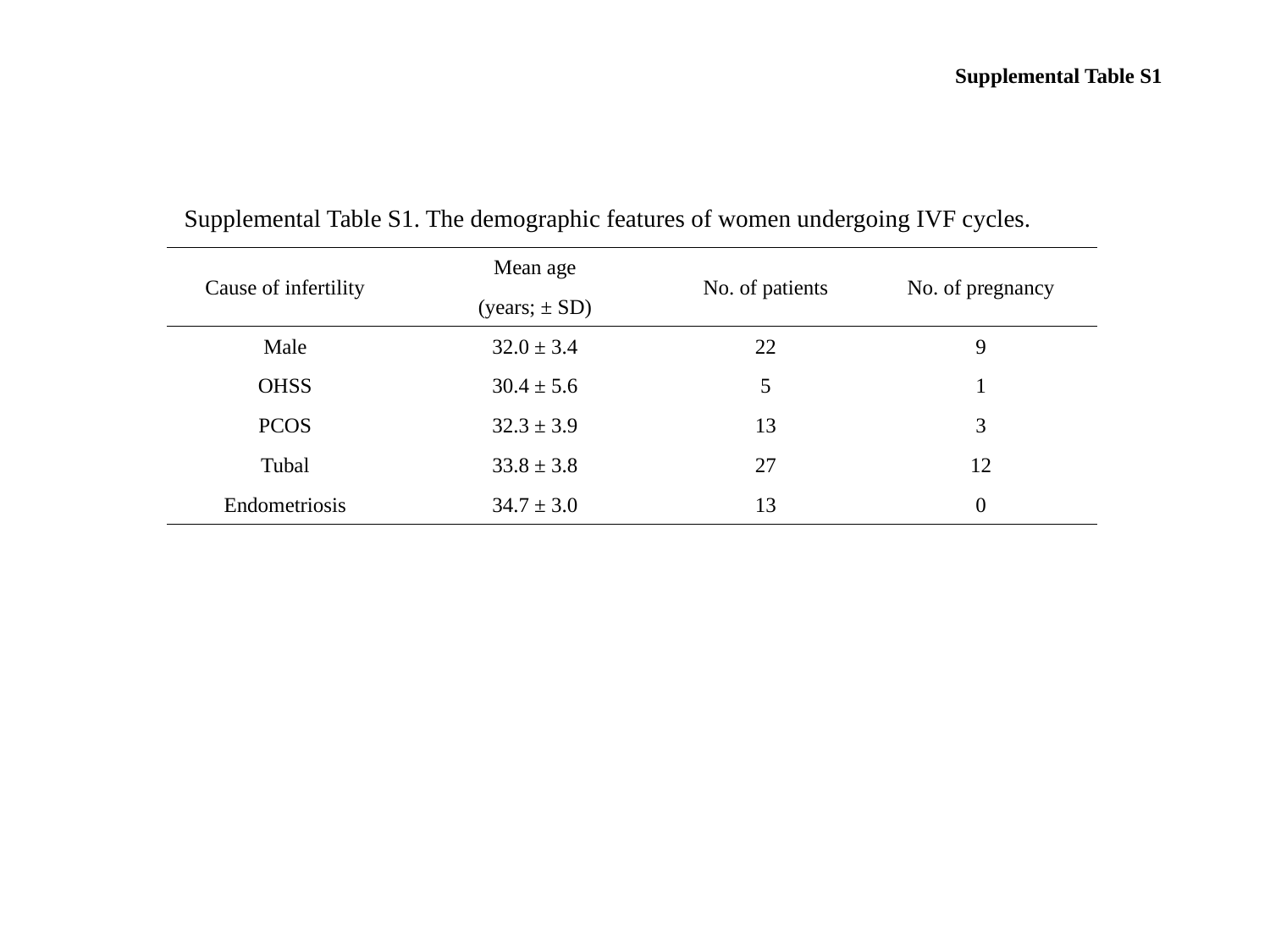

Supplemental Table S1
Supplemental Table S1. The demographic features of women undergoing IVF cycles.
| Cause of infertility | Mean age | No. of patients | No. of pregnancy |
| --- | --- | --- | --- |
| | (years; ± SD) | | |
| Male | 32.0 ± 3.4 | 22 | 9 |
| OHSS | 30.4 ± 5.6 | 5 | 1 |
| PCOS | 32.3 ± 3.9 | 13 | 3 |
| Tubal | 33.8 ± 3.8 | 27 | 12 |
| Endometriosis | 34.7 ± 3.0 | 13 | 0 |

## Slide 2
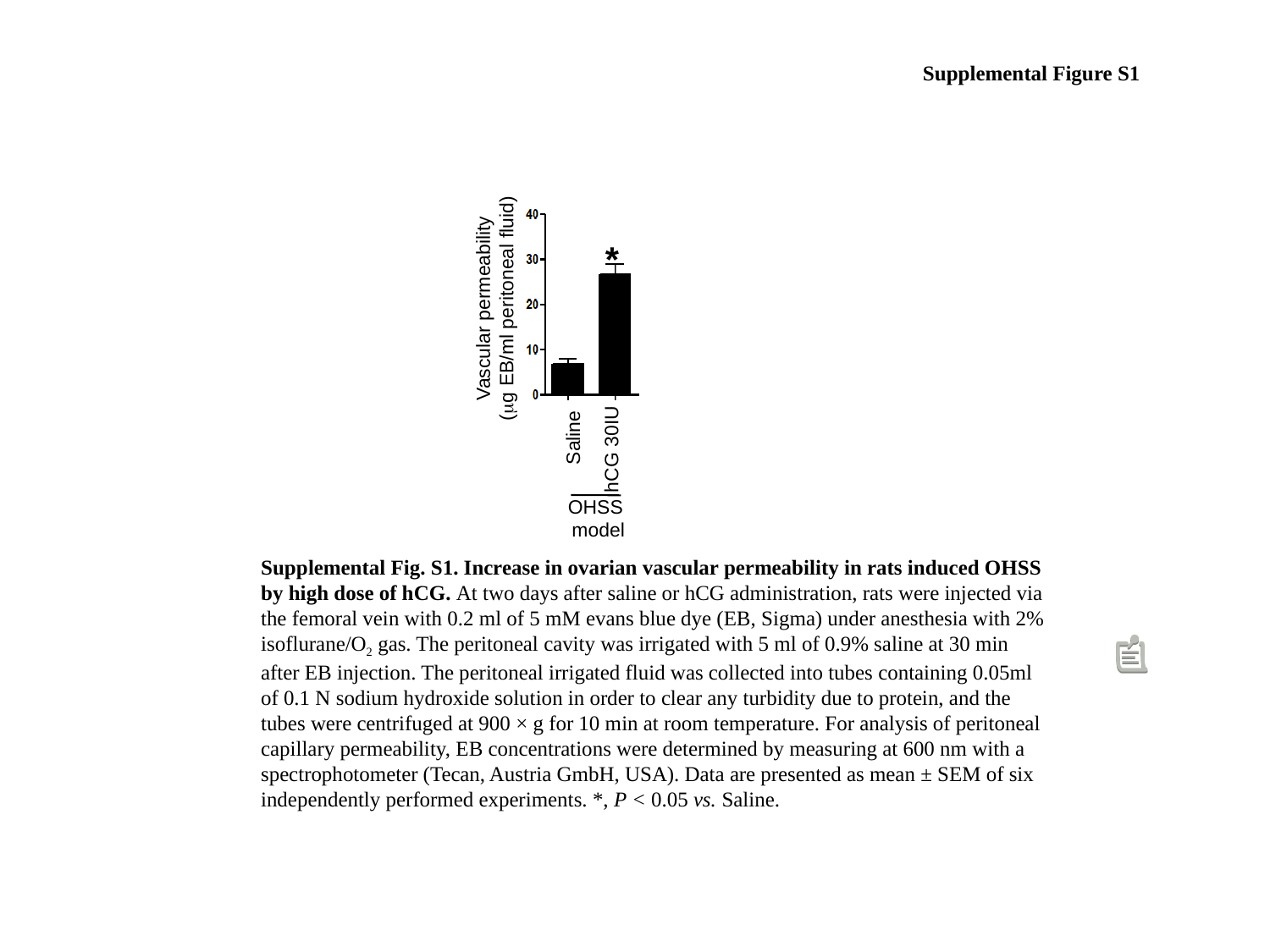

Supplemental Figure S1
*
Vascular permeability
(mg EB/ml peritoneal fluid)
Saline
hCG 30IU
OHSS
 model
Supplemental Fig. S1. Increase in ovarian vascular permeability in rats induced OHSS by high dose of hCG. At two days after saline or hCG administration, rats were injected via the femoral vein with 0.2 ml of 5 mM evans blue dye (EB, Sigma) under anesthesia with 2% isoflurane/O2 gas. The peritoneal cavity was irrigated with 5 ml of 0.9% saline at 30 min after EB injection. The peritoneal irrigated fluid was collected into tubes containing 0.05ml of 0.1 N sodium hydroxide solution in order to clear any turbidity due to protein, and the tubes were centrifuged at 900 × g for 10 min at room temperature. For analysis of peritoneal capillary permeability, EB concentrations were determined by measuring at 600 nm with a spectrophotometer (Tecan, Austria GmbH, USA). Data are presented as mean ± SEM of six independently performed experiments. *, P < 0.05 vs. Saline.
